# Sham Nursing Homes

**Published:** 1902-06-07

**Authors:** 


### The Institutional Workshop.

### Sham Nursing Homes.

*Truth* tells a shocking story of a so-called "nursing home" and of the terrible neglect with which the inmates thereof were treated. Into a little two-story house there were received in one room an old woman disabled with chronic rheumatism and bed-ridden for nine years past, another, over seventy years of age, suffering from bronchitis and asthma and helpless, and a third, said to be ninety years of age, exceedingly feeble in mind as well as body; in another room two women, both over seventy, one subject to giddiness and the other very deaf; in another an old woman given to wandering about at night; in another an old man, apparently in an advanced stage of senile decay and quite childish; in another a woman about fifty, helpless from rheumatism; in another two sisters, one suffering from "shaking palsy" and the other with an ulcerated leg; in another a very old woman quite in her dotage, and a woman with a paralysed arm. There was another patient, whose death led to the investigation, and if she were there at the same time as those above mentioned, that would make thirteen inmates. This "nursing home" was managed by a woman "absolutely illiterate," her husband, and their daughter a little girl of about thirteen. For ten days immediately before Easter a respectable working woman was engaged for further domestic help, but she left because she was horrified at what she saw. In this terrible place "beds seem only to have been made at intervals of several days; bed linen, however foul its condition, to have been changed only at intervals of weeks; patients were left for days without being washed," while the feeding was irregular. But not content with the passive cruelty of neglect, cruelty of a very real and active kind appears to have been practised, presumably in the way of punishment, upon these feeble and half-witted creatures, while some of them were tied down in bed in a manner which would not be permitted in either workhouse or asylum.

Nothing could be worse than the story of neglect, ill-treatment, and positive cruelty which is laid bare in *Truth*, and the question at once arises, What machinery is there by which these things can be prevented? So far as the particular case is concerned we can have but little doubt that, if the details which have been furnished to *Truth* are correct, the owners of such a "nursing home" as is therein described could be punished under the lunacy laws even without any proof of cruelty. The law is perfectly definite as to the illegality of receiving any person of unsound mind for payment except in a licensed house, and from the statements in *Truth* it would appear that several of the inmates of this home came under that category. One "was exceedingly feeble in mind," another "quite childish," another was "quite in her dotage," and her "peculiarity was playing with the fire and asking for her father and mother," while another was "given to wandering about at night, and apparently not quite right in her head." If these people were of "unsound mind" they would evidently come under the beneficent protection of the Commissioners in Lunacy.

That, however, does not really solve the problem, for if one were to eliminate all such mental cases there would probably still remain a large number of people whose relatives, although unwilling to send them to the workhouse, are completely indifferent how they are looked after so that they can be taken off their hands, while undoubtedly there remains another not inconsiderable class of friendless people who, being in receipt of small pensions, or having invested in small annuities drift into "homes" of various degrees and when once there find themselves unable to get away without the assistance of outside friends whom they do not possess. For such unfortunates surely some provision ought to be made, or at least some protection ought to be provided, and we cannot but feel that the general principle that all places where people who are themselves helpless are placed under the care of others, and all places in which any people whatever are placed under restraint should be registered and inspected, is the proper one to go upon.

Prisons, reformatories, workhouses, asylums, nunneries, nursing homes, hydropathic institutions where "rest cures" are carried out, institutions for the cure of inebriety and other drug manias, children's convalescent institutions, all places where infants are received to nurse, even where only one infant is so received, refuges and rescue homes, and even hospitals, form a great group of institutions in which helpless people are left more or less to the mercy of others. Some of these are already inspected in accordance with laws passed specially for the protection of their inmates, others, such as the great hospitals, are under the constant and often very efficient inspection of their committees and subscribers, but some of the institutions above alluded to are under no inspection whatever, and nothing that happens within their walls has any chance of coming to light unless an inquest takes place or a "scandal" arises by which they are forced to wash a little of their dirty linen before the public. There seems no reason for any special legislation in reference to any one class of institution. What we would urge is that the general principle should be adopted that those who take charge of helpless people should declare themselves and submit to registration and inspection all alike, and that "dark places" should no longer be tolerated in this land.

